# Potential genetic modifiers for somatic EGFR mutation in lung cancer: a meta-analysis and literature review

**DOI:** 10.1186/s12885-019-6317-6

**Published:** 2019-11-08

**Authors:** Yue I. Cheng, Yun Cui Gan, Dan Liu, Michael P. A. Davies, Wei Min Li, John K. Field

**Affiliations:** 1 Department of Respiratory and Critical Care Medicine, West China Hospital, Sichuan University, Chengdu, 610041 China; 2 Lung Cancer Research Group, Department of Molecular and Clinical Cancer Medicine, Institute of Translational Medicine, University of Liverpool, William Henry Duncan Building, 6 West Derby Street, Liverpool, L7 8TX UK

**Keywords:** Lung cancer, EGFR mutation, Family history of cancer, Inherited susceptibility, Cancer predisposition genes, EGFR T790 M, BRCA, TP53, DNA repair, Lung cancer aetiology

## Abstract

**Background:**

Accumulating evidence indicates inherited risk in the aetiology of lung cancer, although smoking exposure is the major attributing factor. Family history is a simple substitute for inherited susceptibility. Previous studies have shown some possible yet conflicting links between family history of cancer and EGFR mutation in lung cancer. As EGFR-mutated lung cancer favours female, never-smoker, adenocarcinoma and Asians, it may be argued that there may be some underlying genetic modifiers responsible for the pathogenesis of EGFR mutation.

**Methods:**

We searched four databases for all original articles on family history of malignancy and EGFR mutation status in lung cancer published up to July 2018. We performed a meta-analysis by using a random-effects model and odds ratio estimates. Heterogeneity and sensitivity were also investigated. Then we conducted a second literature research to curate case reports of familial lung cancers who studied both germline cancer predisposing genes and their somatic EGFR mutation status; and explored the possible links between cancer predisposing genes and EGFR mutation.

**Results:**

Eleven studies have been included in the meta-analysis. There is a significantly higher likelihood of EGFR mutation in lung cancer patients with family history of cancer than their counterparts without family history, preferentially in Asians (OR = 1.35[1.06–1.71], *P* = 0.01), those diagnosed with adenocarcinomas ((OR = 1.47[1.14–1.89], *P* = 0.003) and those with lung cancer-affected relatives (first and second-degree: OR = 1.53[1.18–1.99], *P* = 0.001; first-degree: OR = 1.76[1.36–2.28, *P* < 0.0001]). Familial lung cancers more likely have concurrent EGFR mutations along with mutations in their germline cancer predisposition genes including EGFR T790 M, BRCA2 and TP53. Certain mechanisms may contribute to the combination preferences between inherited mutations and somatic ones.

**Conclusions:**

Potential genetic modifiers may contribute to somatic EGFR mutation in lung cancer, although current data is limited. Further studies on this topic are needed, which may help to unveil lung carcinogenesis pathways. However, caution is warranted in data interpretation due to limited cases available for the current study.

## Background

Lung cancer is the most frequently diagnosed cancer and also the leading cause of cancer-related deaths over the world [1]. Despite advances in molecular, pathological and biological research, the pathogenesis of lung cancer has not yet been fully elucidated. Though the predominant risk factor, smoke exposure has widely differing attribution to lung cancer risk across different ethnicities, e.g. over 80% in both males and females in the US [2] and UK [3], but only 57.5% in males and 11.5% in females in China [4]. These significant differences indicate lung cancer aetiology is significantly impacted by other risk factors including inherited susceptibility.

Family history is a simple substitute for genetic susceptibility, easily assessed and less technologically demanding (although limited by societal differences in family size). Multiple epidemiological studies [5–9] demonstrated that family aggregation of malignancies would increase individuals’ lung cancer risk. Some critics argued that the family aggregation of lung cancer might have resulted from a shared environment, such as smoking exposure among family members; because most of the cancers clustering in probands’ families are smoking-related [10], and gene-smoking interactions could not be neglected in lung tumorigenesis [11]. However, evidence on the heritability of lung cancer is also accumulating. Epidemiologically, family history of lung cancer still had a significantly increased risk in never-smoker probands [7], especially in Asians after adjusting confounders including smoking [9, 12]. Genetically, recent genome-wide association studies (GWAS) or sequencing studies of lung cancer unveiled a role of inherited susceptibility component overriding that of smoking behaviour [13]. Some significant risk loci have been found to be genome-wide significantly associated with never-smoker lung cancers [14, 15].

Recently, many potential cancer predisposition genes (CPGs) or susceptibility loci have been revealed by investigating familial lung cancers or lung cancer-clustering families. However, the currently uncovered CPG mutations have been estimated to attribute to only ~ 3% of all cancers [16]. Relevant evidence on CPGs is much more limited compared to somatic mutations in the era of whole-genome sequencing [16, 17].

Since its first discovery in lung adenocarcinoma in 2004, somatic *EGFR* mutation - one of the most important and targetable driver mutations found in non-small cell lung cancer (NSCLC) - has been extensively validated as an effective indicator of sensitivity to *EGFR* tyrosine kinase inhibitors (TKIs), as well as a prognosticator for patients [18]. It is confirmed that exon 19 deletion and L858R point mutation in exon 21 are the most frequently mutated subtypes (the “common mutations”), accounting for 45 and 35% of all the *EGFR*-mutated NSCLC cases, respectively [19]. Rare mutations have less evidence on TKI sensitivity and clinical responsiveness than the common ones, while some consensus has been achieved via individual or selective analysis: mutations occurring within exons 18 to 21 usually confers sensitivity to *EGFR* TKIs, except those within exon 20, such as T790 M and exon 20 insertions [18]. It’s of note, *EGFR*-mutated lung cancers generally have a different epidemiological profile from the *EGFR* wild-type ones, the former more likely to be non-smokers (vs smokers: 37.6%~ 62.5% vs 8.4%~ 35.9% varying by ethnicity), East Asians (vs Westerns: 47.9% vs 19.2% in ADCs) and lung adenocarcinomas (vs SCCs: 47.9% vs 4.6% in Asians) [20–22], which may indicate distinct modulations of relevant variables in tumorigenesis.

Since lung cancers with a family history may indicate a potentially differed genetic background from sporadic cases, it is interesting to investigate if there is a relationship between family history of cancer and *EGFR* mutations in lung cancer patients, both of which participate in tumorigenesis. To date, observational studies reported conflicting relationships, either positive or neutral, between family history and the presence of *EGFR* mutation in lung cancer patients. Given the contradictory epidemiological findings and the potential implication in lung carcinogenesis, we conducted a meta-analysis to pool the risk estimates from previous studies focusing on family history of cancer and somatic *EGFR* mutation; then by a second literature research, we summarized familial lung cancer cases with both potential CPGs and somatic *EGFR* mutation status reported to help to throw a light on this topic.

## Methods

### Meta-analysis of family history on somatic EGFR mutation

We followed the guidelines of the Meta-analysis of Observational Studies in Epidemiology (MOOSE) group for reporting [23]. We searched PubMed, EMBASE, Web of Science and Cochrane Library by using a combination of free text and medical subject heading (MESH) terms related to lung cancer, EGFR and family history (Detailed searching strategies in Additional file 1: Table S1). Hand searching the bibliography of relevant articles was also used.

Our inclusion criteria were as follows: [1] Case-control study, cohort study and other studies of lung cancer patients with *EGFR* mutation status detected/reported [2]; Odds ratios (in case-control studies), relative ratios (in cohort studies) reported relative to a family history of cancer, or of sufficient information to calculate them. If there were several eligible publications derived from the same dataset, the one with the largest sample size was included. Studies with limited or incomplete data including case studies, studies with only *EGFR* mutant cases or incomplete information associating with both *EGFR* mutation status and family history were excluded.

Two independent authors (YIC and YCG) first reviewed all the titles/abstracts to find the potentially related studies, then had a full view of these potentially related studies and selected the eligible studies based on the inclusion/exclusion criteria above. Any discrepancies were resolved by consensus after discussion.

The two reviewers independently extracted information concerning study design, year of publication, study size, study duration, inclusion/exclusion criteria, subjects’ characteristics (age, gender, ethnicity, lung cancer histology, smoking status, family history of lung cancer/other cancer in first/second-degree relatives) at the diagnosis of lung cancer, *EGFR* mutations and detection methods, odds ratio (OR) or risk ratio estimates and the corresponding 95% CIs. The Newcastle-Ottawa scale was used to assess the quality of each included study [24].

Forest plots were generated for meta-analytic estimates by using Mantel-Haenszel (MH) method and random-effects models. Inverse Variance (IV) method was used when only estimates and their standard errors were available in the original studies. Heterogeneity was assessed by using Cochran’s Q and I^2^-statistic. To test the robustness of the estimates, we performed a sensitivity analysis by subgrouping studies. Publication bias was evaluated by applying the funnel plot [24]. We used RevMan 5.3 to perform all the analysis.

### Literature research for underlying mechanisms on somatic EGFR mutation

To further elucidate the topic, we searched PubMed and Web of Science Core Collection using a combination of keywords and/or MeSH terms associating with “lung cancer”, “family history” and “germline mutation” (detailed searching strategies in Additional file 1: Table S2). Then we concluded current papers associating with lung cancer-clustering families which reported their tumour somatic *EGFR* mutation status. Our inclusion criteria were: 1) potential CPGs were investigated and reported in the index case of lung cancer; 2) CPGs were also detected and validated in other family members besides the proband; 3) somatic *EGFR* mutations were reported in the lung tumours in the probands and/or other family members. No ethical approval was needed for the current study.

## Results

### Meta-analysis

**Fig. 1:**
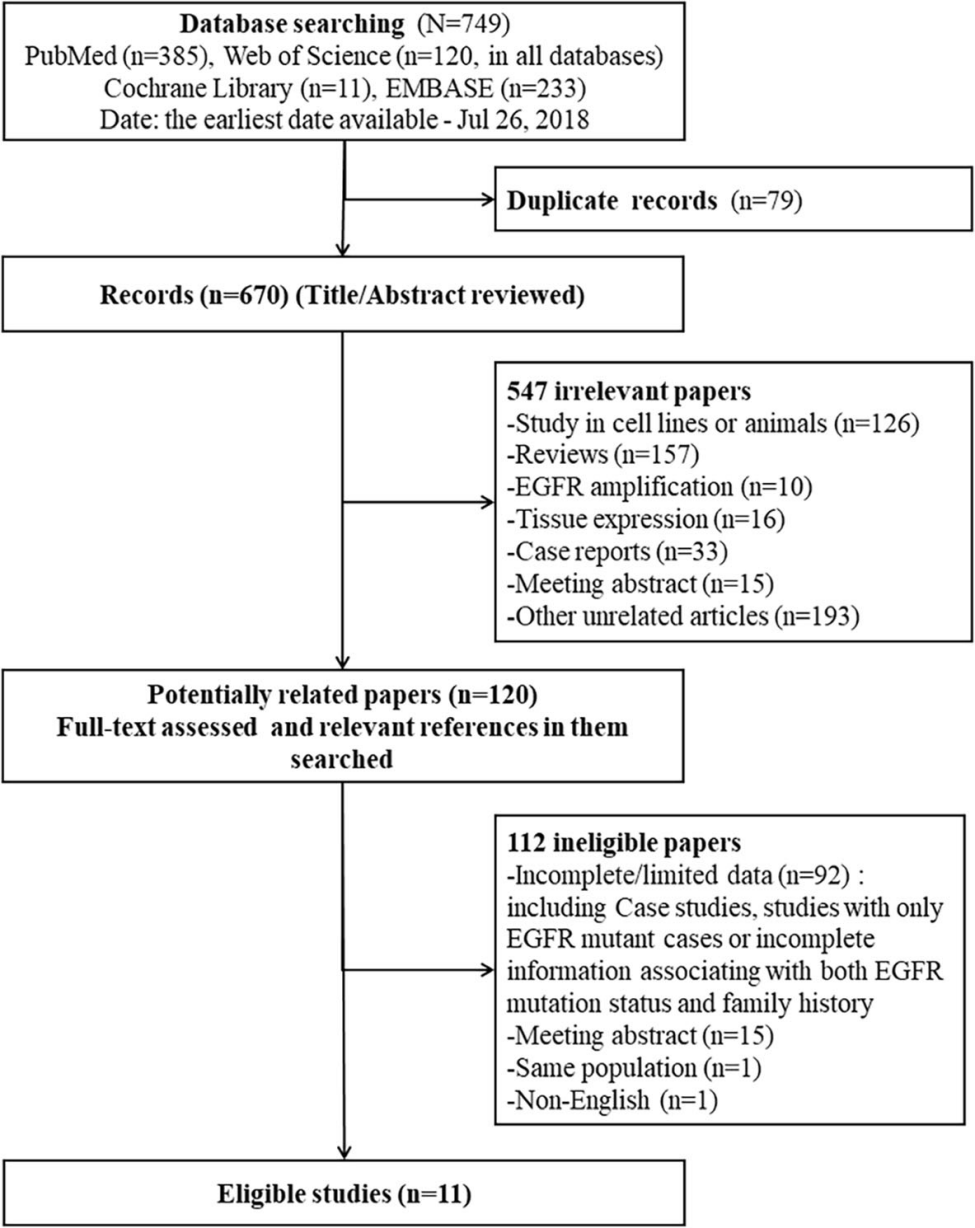
Flowchart of study design for the meta-analysis

After removing duplicates and the initial screening of titles and abstracts, 120 papers were potentially related and undergone through a full-text review. Ninety-two papers had incomplete or limited data, fifteen were meeting abstracts, one was non-English, and another studied the same population as one of the eligible papers (more detailed information in the latter). Thus, 11 original studies were included (Fig. 1). Quality assessment results of each study were shown in Additional file 1: Tables S3-S4.

Table 1 showed the main characteristics of the studies included in the current meta-analysis [25–35]. Ten of them were cohort studies and one was a case-control study. Most of the studies focused on non-small cell lung cancers (NSCLCs) or lung adenocarcinomas (ADCs). There were quite a number of differences in definitions of *EGFR* positive mutation and family history, detection methods and composition of the study population. Due to a very high heterogeneity by pooling all the studies (I^2^ = 78%, *P* < 0.000), we performed the funnel plot and excluded the outlier study by Cheng et al. (2015) [25] in our analysis afterwards (Additional file 1: Figures S1-S2).

**Table 1:** Case-control and cohort studies on family history and *EGFR* mutation status included in the meta-analysis

Study ID	Study /Year	Country	Study design	Sample size	Year at diagnosis	Female	Non-smokers^a^	NSCLC (%)	Lung ADC (%)	Relatives with cancer	Family history (%)	*EGFR* positive mutation definition	*EGFR* mutation (%)	*EGFR* detection method	Detection gene site	Comment
1	Cheng et al. /2015 [25]	Taiwan, China	Case-control	246	2012–2014	89%	100%	100%	93%	1st and 2nd degree	34.6%	NR	40%	PCR direct sequencing	NR	FHLC, FH_All, FH_Other available
2	Gaughan et al./ 2013 [26]	USA	Cohort	230	2004–2011	67%	100%	100%	87%	1st and 2nd degree	56.9%	All mutations	42.6%	PCR direct sequencing	exon18–21	FHLC, FH_All, FH_Other available
3	He et al./ 2013 [27]	China	Cohort	538	2008–2012	44.2%	43.9%	100%	82.0%	1st degree	21.7%	19del, T790 M, L858R	40.9%	ARMS	2 types of deletion of exon 19, T790 M, L858R	FHLC, FH_All, FH_Other available
4	Hsu et al./ 2016 [28]	Taiwan, China	Cohort	1713	2011–2014	54.2%	81.6%	100%	100%	1st degree	7.6%	All mutations	55.8%	MALDI-TOF MS (Mass ARRAY®)	exon 18–21	Only FHLC available
5	Isla et al. / 2016 [29]	Spain	Cohort	830	2007–2012	100%	39.7% ^b^	86.2% ^b^	64.9% ^b^	1st and 2nd degree	50.6%	All mutations	33.9%	PCR sequencing	exon 18–21	FHLC, FH_All, FH_Other available
6	Kawaguchi et al./ 2011 [30]	Japan	Cohort	124	2008–2010	88.1%	100%	100%	96.8%	1st degree	17.4%	19del, L858R	62.7%	PCR-INVADER	exon 18–21	Only FHLC available
7	Kim JS et al./ 2017 [31]	Korea	Cohort	829	2006–2014	35.5%	33.1%	100%	64.8%	1st degree	9.0%	NR	37.2%	PCR direct sequencing	exon 18–21	Only FHLC available
8	Kim SY et al./ 2017 [32]	Korea	Cohort	835	2003–2013	100%	93.4%	100%	89.1%	1st degree	34.1%	19del, L858R, G719X	45.3%	direct sequencing or pyrosequencing	exon 18-21	Only FH_All available
9	Okudela et al./ 2009 [33]	Japan	Cohort	153	2001–2008	49.1%	49.1%	100%	100%	NS	37.3%	19del, L858R	21.6%	PCR direct sequencing	28 exons	Only FH_All available
10	Wang et al./ 2015 [34]	China	Cohort	297	2009–2013	N/A	80.1%	100%	57.9%	1st and 2nd degree	15.2%	19del, L858R, L861Q, S768I, G719S/A/C	45.8%	PCR sequencing	NR	Only FH_All available
11	Zhu et al./ 2014 [35]	China	Cohort	131	2011–2012	43.5%	56.5%	100%	100%	1st and 2nd degree	14.5%	Exon 19 del E746A750, L858R	48.1%	ARMS/ Scorpion	Exon 19 del E746A750, L858R	Only FH_All available

*Abbreviations*: *ADC* adenocarcinoma, *FHLC* family history of lung cancer, *FH_All* family history of all cancers, *FH_Other* family history of other cancer (except lung cancer), *NSCLC* non-small cell lung cancer, *PCR* polymerase chain reaction, *USA* United States of America

^a^Exposure < 100 cigarettes in one’s life time

^b^in total 1762 lung cancer cases, of which 830 cases had EGFR mutation status available

**Fig. 2:**
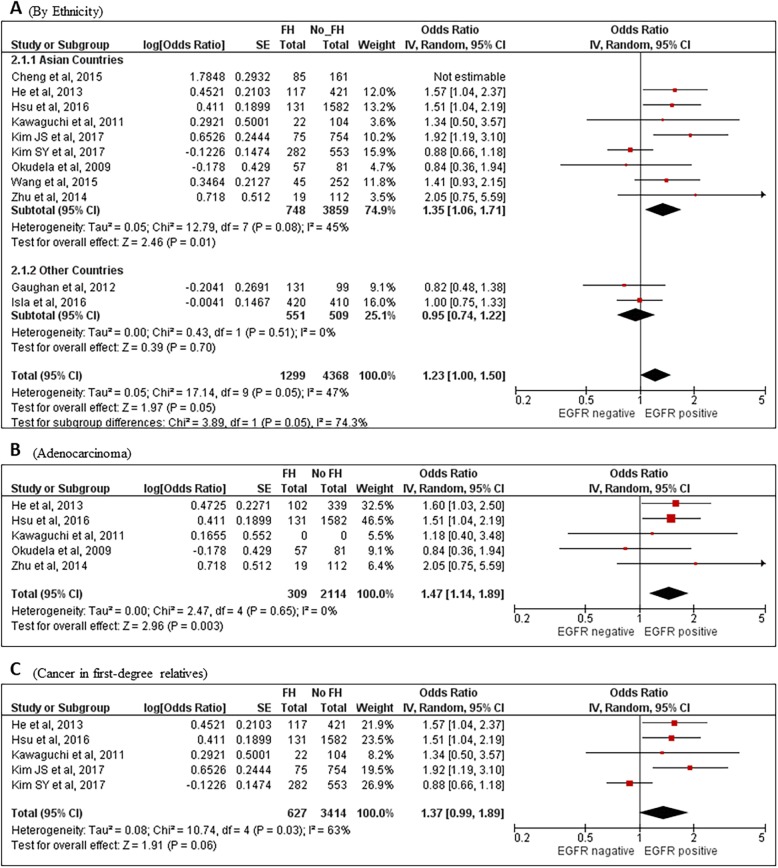
Forest plots for family history of any cancer and the risk of EGFR positive mutation. **a** Overall and by country: **b** in lung adenocarcinoma patients; and **c** patients with family history of any cancer in first-degree relatives. FH, family history; IV, Inverse Variance method. CI, confidence interval

Figure 2 provided the “overall” likelihood of *EGFR* mutation status in lung cancer patients with family history of any cancer (FH_Any) compared to those without from the remaining ten studies. “Overall” estimates of FH_Any here referred to the total effects by pooling the studies without differentiating family history of all cancers, lung cancer or other non-lung cancers. There was a marginal significance (OR = 1.23[1.00–1.50], *P* = 0.05) with an intermediate heterogeneity among studies (I^2^ = 47%, *P* = 0.05). When restricted to Asian countries (eight studies), the difference became significant (OR = 1.35[1.06–1.71], *P* = 0.01) (Fig. 2a). In lung adenocarcinoma (ADC) patients with FH_Any, *EGFR* was more likely mutated than those without (OR = 1.47[1.14–1.89], *P* = 0.003) (Fig. 2b). Marginal significance was also observed in patients with cancer in their first-degree relatives than their FH_Any-absent counterparts (OR = 1.37[0.99–1.89], *P* = 0.06) (Fig. 2c). However, there were no significant findings when limiting patients to females, never-smokers or those having FH_Any yet with both their first- and second-degree relatives included, possibly due to much less data in these subgroups.

**Fig. 3:**
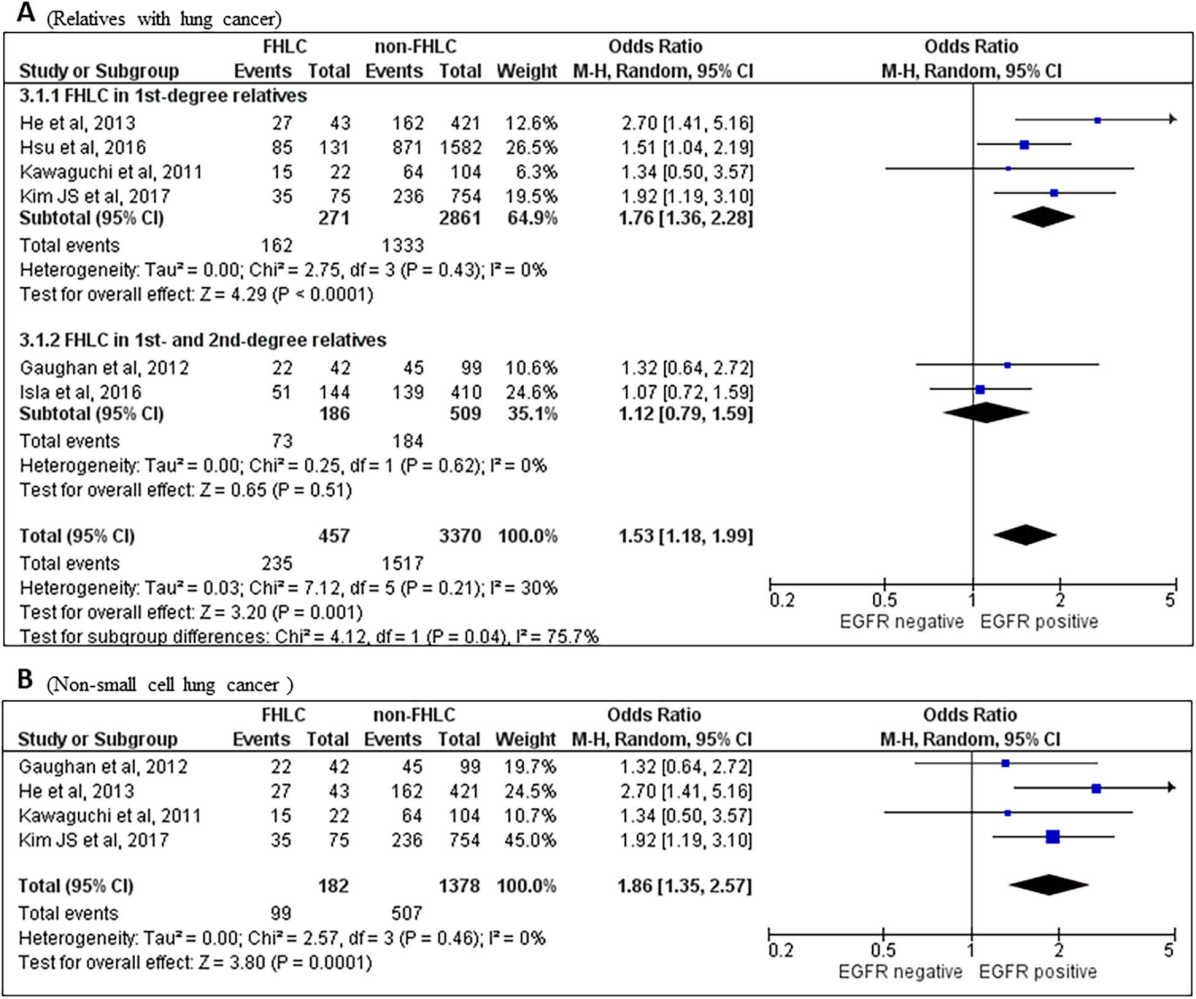
Forest plots for family history of lung cancer and the risk of EGFR positive mutation. **a** Overall and according to relatives and **b** in non-small cell lung cancer patients. FHLC, family history of lung cancer; M-H, Mantel-Haenszel method; CI, confidence interval

There was a significantly higher proportion of *EGFR* mutation in patients with family history of lung cancer (FHLC) than those without (OR = 1.53[1.18–1.99], *P* = 0.001) (Fig. 3a), including in analyses limited to those who had lung cancer in their first degree relatives (OR = 1.76 [1.36–2.28], *P* < 0.0001) (Fig. 3a). The association between *EGFR* mutation and FHLC-positive cases remained significant when limited to those diagnosed as NSCLCs (OR = 1.86[1.35–2.57], *P* = 0.0001) (Fig. 3b). Only one study reported data of *EGFR* mutation specifically in ADC patients with FHLC, which indicated a significantly higher possibility of mutation than those absent of FHLC (OR = 1.51[1.04–2.19], *P* = 0.03). The association between the two variables was not altered greatly if only Asian patients were included (Data not shown since neither of the two excluded non-Asian studies showed significant results). Further subgroup analysis of *EGFR* mutation status in patients with/without FH of all cancers or other non-lung cancers did not demonstrate any remarkable difference between subgroups tested (Data not shown).

### Results of the second literature search

In total, there were 41 lung cancer cases in 29 families eligible for our second analysis (Tables 2 and 3**)**. The median onset age was 57 years-old (range 22–78). Females (31/41, 75.6%) and never-smokers (24/41, 58.5%) predominated in the curated cases. Almost all (35/41, 85.3%) of the histology in lung cancer patients were ADCs; the remaining five patients were diagnosed as NSCLCs (uncategorized) and another one was SCC. In this dataset, there were eight White and seven Asian families. Five of the White families inherited the *EGFR* gene; while CPGs in the Asian families were more scattered (but report bias could not be excluded here).

**Table 2:** Lung cancers with germline cancer predisposing genes detected and somatic EGFR mutation information in lung cancer-clustering families

Germline genes or loci	Germline mutations	Case #	Family #	Relation	Sex	Age	Ethnicity	Smoking	Histology	Somatic EGFR mutation	Comment	Ref.
*EGFR*	p.T790 M	1	1	Proband	M	50	White	S	5 × ADCs	2 × L858R, 1 × 19del, 2 × WT	Mother (F, 62, BAC); Maternal grandfather (M, 72, BAC); Maternal great uncle (M, 60s, BAC); Brother (51, male, multi-nodules) and Sister (48, female, unaffected) carried germline *EGFR* p.T790 M	Bell, et al. 2005 [36]
		2	1	Brother	M	55	White	S	ADC	G719A		
*EGFR*	p.T790 M	3	2	Proband	F	72	NR	NS	1 × ADC + 1× BAC + 1 × LCC	3 × WT	Sister affected with lung cancer	Prudkin, et al. 2009 [37]
*EGFR*	p.T790 M	4	3	Proband	F	62	NR	NS	ADC	WT	Mother had lung cancer	Prudkin, et al. 2009 [37]
*EGFR*	p.T790 M	5	4	Proband	F	72	White	NS	ADC	19del	Inconsistent records in the pedigree (aged 73 and having SCC)	Tibaldi, et al. 2011 [38]
		6	4	Sister	F	74	White	NS	NSCLC	WT	Exon 20 was not examined due to insufficient tumour tissue.	
*EGFR*	p.T790 M	7	5	Proband	F	70	NR	S	ADC	WT	Father (M, 60s, smoker, lung cancer); brother (male, 62, smoker, throat cancer); Proband had somatic *K-RAS* mutation.	Thomas, et al. 2013 [39]
*EGFR*	p.T790 M	8	6	Proband	F	58	NR	S	ADC	L858R	Mother (female, 70s, non-smoker, BAC); brother (male, 45, ADC), brother (male, 51, non-smoker, bilateral lung nodules of uncertain cause at follow-up)	Thomas, et al. 2013 [39]
*EGFR*	p.T790 M	9	7	Proband	F	29	White	LS	ADC	L858R	Proband also had multiple lesions including AAH, AIS and MIA. Fourteen carriers with known, obligate or assumed mutations in the family pedigree; in these carriers, 4 had lung cancer. In Five unaffected mutation carriers, four had multiple nodules and the other one had single sub-cm solid nodule.	Gazdar, et al. 2014 [40]
*EGFR*	p.T790 M	10	8	Proband	F	44	NR	NS	7 × ADCs	4 × L858R, 2 × 19del, 1 × WT	The *EGFR* wild-type ADC had somatic *ARID1A* p.K1938 N. Family history of breast and ovarian cancer in maternal relatives (2nd-degree); germline *BRCA2* p.L459S variant of uncertain significance detected. Mother with metastatic ADC (germline T790 M carrier, unknown age, *BRCA1/2* not detected); Daughter carried germline T790 M.	Yu, et al. 2014 [41]
*EGFR*	p.T790 M	11	9	Proband	F	34	White	NS	ADC	L858R	Family history of lung and other cancers (paternal relatives); no germline EGFR T790 M status available in other members	Lou, et al. 2016 [42]
*EGFR*	p.R776H	12	10	Proband	F	57	White	NS	NSCLC	G719A	NSCLC with squamous component inside. Only a brother detected and did not carry the germline R776H mutation.	Van Noesel, et al. 2013 [43]
		13	10	Daughter	F	36	White	NS	SCC	G719S		
*EGFR*	p.V769 M	14	11	Proband	M	57	Jewish	S	5 × ADCs	2 × G719A, 2 × (G719C + S768I), 1 × G719S	Family history of other cancers (breast and ovarian cancers in the 2nd-degree maternal relatives), did not examine *BRCA1/2*; the proband also present several small lung nodules in the lung postoperatively	Hellman, et al. 2017 [44]
*EGFR*	p.V843I	15	12	Proband	F	70	Asian	UK	3 ADCs + 4 BACs + 3 AAHs	3 × L858R (1 ADC, 1 BAC, 1 AAH), 2 × L861Q (2 ADCs)	Other 5 lesions haven’t been examined. Father and a brother died of lung cancer. A healthy sister and another unaffected brother carried the germline V831I mutation.	Ikeda, et al. 2008 [45]
*EGFR*	p.V843I	16	13	Proband	F	78	Asian	UK	ADC	L858R	Aunt had ADC at 70 (germline not examined). A nephew had non-Hodgkin’s lymphoma at 12 (germline V843I negative). A healthy daughter carried germline V843I mutation.	Ohtsuka, et al. 2011 [46]
		17	13	Mother	F	70	Asian	UK	ADC	L858R		
		18	13	Brother	M	41	Asian	UK	ADC	L858R		
*EGFR*	p.V834 L	19	14	Proband	F	57	Surinam	S	ADC	L858R	A daughter carried germline V834 L; Father died of massive hemoptysis of unknown cause.	Van der Leest, et al. 2018 [47]
		20	14	Brother	M	57	Surinam	S	NSCLC	L858R		
		21	14	Sister	F	46	Surinam	NS	NSCLC	L858R		
		22	14	Daughter	F	42	Surinam	NS	NSCLC	L858R		
*HER2*	p.G660D	23	15	Proband	F	44	Asian	LS	Multi-ADCs	WT	HER2 Family history of lung cancers among multiple maternal members; Daughter with germline G660D, and CT showed multiple GGNs in bilateral lungs at 30 (light smoker).	Yamamoto, et al. 2014 [48]
		24	15	Mother	F	74	Asian	NS	Multi-ADCs	WT		
*MET*	p.N375K	25	16	Proband	F	75	Asian	NS	ADC	L858R	Another sister (never-smoker) clinically diagnosed with lung cancer at 80.	Tode, et al. 2017 [49]
		26	16	Sister	F	63	Asian	NS	ADC	19del		
		27	16	Sister	F	63	Asian	LS	ADC	L858R		
*CHEK2*	p.R474C (homozygous)	28	17	Proband	M	60	Asian	NS	Multi-ADCs,	NR	Proband: colon and prostate cancer affected. Father (60 year): prostate and gastric cancer; Mother (79 year): solitary lung cancer; A son (1 year 10 moths): neuroblastoma.	Kukita, et al. 2016 [50]
		29	17	Sister	F	60	Asian	NS	7 ADCs,	L858R or 19del	Uterine myoma and breast cancer affected	
*BRCA2*	c.9641insT	30	18	Proband	M	43	White	NS	ADC	Exon 20ins	Family history of breast cancer in maternal relatives and lung cancer in maternal grandfather (never smoker)	Marks, et al. 2008 [51]
*BRCA2*	c.8867del5	31	19	Proband	M	74	White	LS	ADC	19del	Family history of breast or ovarian cancers in daughter, mother and maternal aunt. Daughter carried germline *BRCA2* c.8867del5 mutation.	Marks, et al. 2008 [51]
*TP53*	p.R273H	32	20	Proband	F	34	NR	NS	ADC	19del	Proband: breast cancer affected at 30 (somatic *HER2*+, *EGFR*-). Mother with bilateral breast cancer at 35; Sister 1 with breast liposarcoma at 26 (germline *TP53* p.R273H); Sister 2 with breast cancer at 33 (germline *TP53* p.R273H); maternal grandmother with breast cancer at early 40s; Brother unaffected (germline *TP53* p.R273H); Sister 3 without germline *TP53* mutation; all without germline *BRCA1/2*.	Bemis, et al. 2007 [52]
*TP53*	p.G245S	33	21	Proband	F	43	Hispanic	NS	ADC	L858R	Concurrent somatic *HER2* p.S310F. Germline *BRCA1/2* negative. Affected breast cancer at 44, gluteal schwannoma at 46 and atypical leiomyoma. Sister and Aunt with breast cancer at 40s; Cousin with brain tumour at a young age; Mother with leukaemia.	Jia, et al. 2014 [53]
*TP53*	exon 19 deletion	34	22	Proband	F	51	White	NS	ADC	L858R	Proband: bilateral breast cancers and malignant fibrous histiocytoma affected. Mother, maternal aunts, two first cousins and maternal grandmother died of early-onset cancers (< 60 years)	Michalarea, et al. 2014 [54]
*TP53*	p.H179Y	35	23	Proband	M	55	NR	NS	ADC	19del	T790 M mutation (post-TKI) detected; No somatic alterations on *HER2*, *PI3KCA*, *BRAF*, *KRAS* or *ALK* genes. Descendants affected with unusual childhood tumours.	Ricordel, et al. 2015 [55]
*TP53*	p.R273H	36	24	Proband	F	57	NR	NS	ADC	L858R	Affected with breast cancer as well. No somatic alterations on *HER2*, *PI3KCA*, *BRAF*, *KRAS* or *ALK* genes. Daughter affected with corticosurrenaloma.	Ricordel, et al. 2015 [55]
*TP53*	p.G245S	37	25	Proband	F	30	NR	NR	ADC	19 del	Daughter affected with sarcoma at 10. Another two children are carriers.	Pathak, et al. 2018 [56]
*TP53/CDH1*	p.R196^a^; CDH1 p.N570=	38	26	Proband	F	26	Hispanic	NS	3 ADC	1 × 19del	Proband: the other two ADCs with EGFR amplification and *PIK3CA* p.E545K. Intra-alveolar lung tumour spread with *K-RAS* p.G12C + *BRAF* p.L597 V; Osteosarcoma affected at 12 (somatic *PIK3CA* p.E545K + *K-RAS* p.G12S + *CDH1* p.A617T). Mother with breast cancer at 32; Maternal Uncle with facial and orbitary chondrosarcoma at 14 and diffuse gastric cancer at 24; Maternal Uncle with anaplastic astrocytoma at 13; Maternal Cousin with diffuse gastric cancer at 36 (germline *CDH1* p.Leu721Val); Maternal Cousin with *EGFR*-mutated lung cancer at 26; Maternal Grandmother with breast cancer at 50, melanoma at 44 and colon cancer at 50; Paternal Aunt with breast cancer at 48.	Cardona, et al. 2018 [57]
*TP53*	p.R248W	39	27	Proband ^a^	F	34	NR	NS	2 ADC	1 × exon 20ins	The other ADC had *HER2* (amplification + p.V659E). Affected bilateral breast ductal carcinoma in situ at 29. Did not report family history.	Serra, et al. 2013 [58]
*TP53/PMS2*	p.V157D/ p.R20Q	40	28	Proband	M	22	Asian	NR	ADC	19del	Family history of a wide variety of tumours (including breast cancer, lung cancer) among family members (affected <=54, half of them < 31 years); father (31, died of colon cancer, *K-RAS* p.G12D mutation in colon tumour) carried the two germline mutations	Wang, et al. 2014 [59]
*APC*	c.TCA1110TGA	41	29	Proband	F	43	Asian	NS	ADC	WT	No germline MYH mutations; Somatic *K-RAS* and p53 wild-type; amplification of three regions 5p, 8q, and 12q14-12q2; affected with FAP at 26, duodenal adenomas at 33. Father with FAP; Son with FAP and medulloblastoma; Paternal great aunt with FAP (whose son was affected with FAP and desmoid tumour, granddaughter with FAP, gastric and thyroid cancer).	Shinmura, et al. 2008 [60]

*Abbreviations*: *AAH* atypical adenomatous hyperplasia, *ADC* adenocarcinoma, *AIS* adenocarcinoma in situ, *BAC* bronchioloalveolar carcinoma, *F* female, *FAP* familial adenomatous polyposis, *GGN* ground-glass nodule, *LS* light smoker, *M* male, *MIA* minimally invasive adenocarcinoma, *NS* never smoker, *NSCLC* non-small cell lung cancer, *S* smoker, *SCC* squamous cell lung cancer, *WT* wild type. Genes were noted as *italics*

^a^The original publication did not report family history of cancer of the index case, but we included it here due to its diagnosis of familial Li-Fraumeni Syndrome

**Table 3:** Clinical characteristics of familial lung cancer cases curated in Table 2

Characteristics	All	Germline EGFR carrier
Case No.	41	22
Age at diagnosis		
Median (range)	57 (22-78)	57 (29–78)
Gender		
Male	10 (24.4%)	5 (22.7%)
Female	31 (75.6%)	17 (77.3%)
Smoking Status		
Smoking ^a^	11 (26.8%)	8 (36.4%)
Non-smoking	24 (58.5%)	10 (43.5%)
Not reported	6 (14.6%)	4 (18.1%)
Family No.	29	14
Ethnicity	29 (100.0%)	14 (100.0%)
White	8 (27.6%)	5 (35.7%)
Asian	7 (24.1%)	2 (14.3%)
Other	4 (13.8%)	2 (14.3%)
Not reported	10 (34.5%)	5 (35.7%)
Multiple lung tumours	10 (34.5%)	6 (42.9%)
Lung tumour No. ^b^	≥78	47
Histology (by tumour)		
Adenocarcinoma	72 (~ 92.3%)	41 (87.2%)
Other ^c^	6 (~ 7.7%)	6 (12.8%)
Somatic co-occurring *EGFR* mutation status		
Mutated^d^	54 (69.2%)	33 (70.2%)
L858R	26 (48.1%)	19 (57.6%)
Exon 19del	11 (20.4%)	4 (8.5%)
Other	12 (22.2%)	10 (30.3%)

^a^Including both light smokers and smokers in Table 2

^b^Detailed number of lung tumours were not available in some cases diagnosed with “multiple lung cancers”, thus we recorded their number as ≥2 per case. The tumour number in Case #9 in Family #7 was recorded as one due to incomplete information regarding other pre-cancerous and pre-invasive lesions in the lung

^c^Including five non-small cell lung cancers and one squamous cell lung cancer

^d^We recorded the mutated tumours in Case #29 in Family #17 as two (one L858R and the other 19del) due to no detailed information. Other mutations included G719C/S/A and exon 20 insertions

Fourteen families (of 29, 48.3%) reported germline *EGFR* mutations, and eight of them carried the T790 M mutation [36–42]. Other germline *EGFR* mutations included R776H [43] and V769 M [44] in exon 20, and V834 L [47] and V843I [45, 46] in exon 21. Nine index patients (of 29, 31.0%) had inherited *TP53* mutations, among whom two had another concurrent germline mutation, respectively (Case No. 38 and Case No. 40) (Table 2).

Ten (of 29, 34.5%) families had multiple lung cancers diagnosed or multiple lung nodules found in the probands or among their family members, which made in total over 78 tumours across the dataset. Specifically, six families (of 14, 42.9%) with multiple lung lesions harboured inherited *EGFR* mutations.

Among all the 78 tumours, fifty-four (~ 69.2%) of these tumours carried a subsequent positive somatic mutation. In the subgroup of inherited *EGFR* mutations, a secondary activating mutation occurred in 70.2% (33/47) of the germline *EGFR* mutation carrier lung cancer cases; similarly, in lung cancers diagnosed in germline T790 M mutation carriers, the proportion of a secondary activating mutation was 73% [40]. Both of the concurrence rates above were higher than that reported in the sporadic NSCLCs (10%~ 35%) [61]. About a half of them were *EGFR* L858R mutation; 48.1% (26/54) in all the curated inherited lung cancers and 57.6% (19/33) in the inherited *EGFR* subgroup (Table 3).

## Discussion

Based on our study, a significant association between family history of malignancy and *EGFR* mutation in lung cancer has been observed in Asians, patients diagnosed as ADCs/NSCLCs or those with lung cancer-affected (first-degree) relatives. Individuals with family history of lung cancer among first-degree relatives have a high risk of lung cancer, bearing an OR ranging 1.51–1.63 after adjustment of other potential confounders [7, 8]; Asians have the highest risk compared to the White and Black/African Americans (adjusted OR: 2.38, 1.46 and 1.67, respectively) [8]. Besides, somatic *EGFR* mutations occur more frequently in Asians, ADCs, females and never-smokers [20–22], a preferential subpopulation partly overlapping with that in our findings.

Family history is a substitute for inherited susceptibility. Recent studies have revealed some germline loci significantly contributing to the likelihood of *EGFR* mutation in lung cancer, e.g. 3q28 (rs7636839, *TP63*), 5p15.33 (re2736100 and rs2853677, *TERT*), 6p21 (rs2495239, *FOXP4*; rs3817963, *BTNL2*; rs2179920, *HLA-DPB1*), 6q22.2 (rs9387478, *ROS1/DCBLD1*) and 17q24.3 (rs7216064, *BPTF*) in Asians [62–64]. These findings suggest underlying genetic modifiers responsible for a predisposition to somatic *EGFR* mutation in lung cancer. Thus, it will be interesting to investigate the potential role of CPGs in the pathogenesis of somatic *EGFR* mutation in lung cancer.

We summarized the potential CPGs and mutated sites reported in familial lung cancers where somatic *EGFR* mutation status was available. Almost all the publications reported the predisposition genes by case-studying one or several lung cancer-clustering families. Some lung cancers complicated or fell within the spectrum of clinical manifestations of familial cancer syndromes. Though limited, the curated data may help to shed light on genetic mechanisms in modifying somatic alterations.

About a half of the families in our curated dataset have reported germline *EGFR* mutation among family members, mostly T790 M and in the White families. Germline *EGFR* mutations are very rare, less than 1/7500 (0.01%) in the general population [40]; the proportion is higher in sporadic lung cancers, namely 1/555 (0.18%) of lung ADCs from TCGA (mostly White) [65] and 14/12,833 (0.11%) of Chinese lung cancers [66]. In two small datasets of familial cases lately, none of the patients has been detected as positive [67, 68].

As the most reported germline mutation, T790 M accounted for 1.0% (5/503) in *EGFR*-mutated lung cancers from the US. Comparably, the proportion of germline T790 M mutation was much lower in Asians, i.e. 0/627 in Japanese NSCLCs [40] and 1/12,833 in Chinese lung cancers [66], notwithstanding their substantially higher somatic *EGFR* mutation rate in the tumours. Therefore, there is inherited susceptibility difference across ethnicities, which may explain the potentially preferential distribution of cancer predisposition genes in our curated families.

Most of the cases with inherited *EGFR* mutation in our investigation had concurrent activating mutations in their tumours. Generally, the germline *EGFR* mutations reported could be oncogenic if alone [42–44, 46, 69]; and the growth potential would be enhanced dramatically when co-occurring with a secondary activating mutation [42–44, 46, 69], which may indicate a ‘second-hit’ proliferative advantage in the tumours [42, 70]. Second somatic activating mutations non-randomly occurred in cis to the inherited mutations [36, 43, 46, 47]. Specifically, *EGFR* T790 M, the mutation responsible for over 50% of the acquired resistance post-TKI in *EGFR*-mutated lung cancers [61], emerges in cis with the initial drug-sensitizing *EGFR* mutation in the tumour as well [71]. T790 M has a modest oncogenic effect, which may be the explanation that it is tolerated in humans as a germline mutation [72]. In a cis configuration with the activating mutation, T790 M mutation could dramatically enhance *EGFR* catalytic activity, and thus, achieve a significant gain of function in transformation and tumour aggressiveness [36, 71, 72]. The increased proliferative advantage of the dual mutations has been observed in experimental conditions [73] as well as in clinical practice [74]. The evidence concerning the mechanisms of the mutual interactions between concurrent double mutations is limited. Presumably, the germline mutation carrier may more likely predispose to lung cancer or develop in a more aggressive nature following the subsequent second somatic mutation; and of note, it is not rare that these carriers have multiple apparently independent lung cancers or lung nodules, the later possibly associated with precancerous or pre-invasive lesions [40].

The distribution of secondary somatic mutations was not typically concordant across family members or multiple lung cancers in the same patient, similar to a previously reported small familial cohort [28]. However, there are some exceptions in our study. Familial cases with germline mutation V769 M had the somatic mutation at codon 719 [44]. Specifically, energy balance could be an explanation for the phenomenon: V769 M alone or with secondary mutations (except L858R) cost less energy to keep *EGFR* in the activated configuration than in the inactivated state, thus causing activation of *EGFR* [44]. For this reason, V769 M is more likely to combine with other mutations than L858R [44], which might be indirectly evidenced by the case reports from the COSMIC database where no concurrent V769 M and L858R mutations have been recorded yet [75]. The other three families, germline R776H with a somatic mutation at codon 719 [43], and germline V834 L [47] and V843I [46] with somatic L858R among different family members, also caught our attention. However, in the records from COSMIC, no exclusive relations between these double mutations have been observed in the R776H, V834I or V843 L-mutated cases (but note that the origin of these mutations in COSMIC – somatic or germline - are mostly unknown and the sample size was small) [75]. Thus, coincidence could not be excluded here. Whether some other precise mechanisms are associated with the preferential combinations in dual/multiplex mutations, like energy balance, and how they function, have yet to be clarified.

Most of the remaining families had germline CPGs functioning in response to DNA damage or regulating DNA repair pathways, including *BRCA2* [51], *CHEK2* [50], *TP53* [52–59] and *PMS2* [59]. Carriers of these CPGs are vulnerable to familial cancers or inherited cancer syndromes, which could overlap with lung cancers, i.e. *BRCA2* in hereditary breast/ovarian cancer [51], *APC* in familial adenomatous polyposis [60] and *TP53* in Li-Fraumeni Syndrome [52–59]. Somatic *EGFR* mutations in these lung cancers are tentatively deletions or insertions (Table 2). Remarkably, these cases are affected with multiple-site lesions. In a recent analysis of germline sequencing data of 555 lung adenocarcinomas from TCGA, the authors found about 2.5% of the lung cases carried the germline mutations that could be linked to inherited risk [65]. Most of them are in DNA repair pathways, including *ATM* (7, 1.3%), *TP53* (4, 0.7%) and *BRCA2* (1, 0.18%) [65], which are closely associated with familial cancer syndromes. What’s more, individuals carrying these predisposing genes or cancer syndromes would have an increased risk of lung cancer [76–78].

Somatic driver mutations, including *EGFR* mutations, occur early in lung cancer evolution, and these early-occurring mutations tend to be histological-subtype-specific [79]. Generally, squamous-cell lung cancer harbours remarkably more clonal mutations (relating to early driver mutations) than lung adenocarcinoma due to differences in smoking behaviours. In never-smoker NSCLC females, somatic *EGFR* mutations are associated with increased exposure to environmental tobacco smoke [80]. Both active and passive smoking are exogenous insults and could result in genotoxic damage which can be enhanced when the endogenous DNA repair system is compromised. Thus, there may be a genetically definable subset of lung cancer patients harbouring germline mutations involved in the dysfunction of DNA repair system, where genomic instability may be a potential risk modifier for *EGFR* mutation in lung tumour.

*BRCA1/2*, the genes responsible for double-strand break repairing, had a significantly lower expression due to its promoter hypermethylation in lung adenocarcinoma [81], potentially mediating genetic instability in lung tumorigenesis. Women with breast cancer have an increased risk of synchronous lung cancer (Hazard Ratio: 5.86 in ages 40–69) and vice versa [82]. Members in the hereditary branch of families of patients eligible for *BRCA* test are at high risk of lung cancer, with an odds ratio of 4.5 compared to those belonging to the non-hereditary branch [78]. Twelve families in our curated dataset reported family or personal history of breast or ovarian cancer, five of which had germline *BRCA1/2* detected, and three probands had positive *BRCA2* germline mutations. All the three index cases were ADCs yet with somatic *EGFR* mutated in different codons. Some small subsets investigated the association between germline *BRCA1/2* mutation and *EGFR*-mutant lung cancer, but didn’t have positive findings due to the rare frequency of the *BRCA1/2* germline mutations [51].

Nine index patients with germline *TP53* mutation in our dataset complexed with Li-Fraumeni Syndrome, which is associated with multiple, often rare, cancers. The nine index families presented early onset of cancer at multiple sites across the families, which was typically consistent with the clinical features of Li-Fraumeni Syndrome. The median age of the index patients was 34 years old at the diagnosis of lung cancer (range 22–57), and females (7/9) and never-smokers (7/9) predominated. One case had concurrent somatic *EGFR* activating mutation and *HER2* point mutation [53]. Usually, these two driver mutations occur mutually exclusively [83]; but in this case may result from defective DNA repair due to *TP53* mutation.

Somatically, *HER2/ERBB2* is mutated in 2–4% of all NSCLCS, of which 80%~ 100% are insertions in exon 20 [83]. Germline mutations in *HER2* are also extremely rare: only one in 12,833 Chinese lung cancer patients has been identified by targeted next-generation sequencing. Yamamoto et al. reported the germline mutation *HER2* G660D in the index family along with a germline *HER2* V659 M mutation detected in a sporadic lung ADC [48]. Mutations on the transmembrane domain could favour kinase activation and *ERBB2* dimerization thus stimulating the *MEK/ERK* signalling [84]. Both G660D and V659E are located at the transmembrane domain. Their mutant proteins are more stable than the wild-type and possess an oncogenic potential by activating *Akt* and *p38*, thus facilitating cell growth and survival [48]. *MET* and *EGFR* are mutual complements, which activate the *PI3K-AKT* pathway by interacting with ERBB3; therefore, the inactivation of *MET* by its heterozygous germline mutation could complementarily enhance the *EGFR-ERBB3-PI3K* axis [49]. The oncogenic stress may explain the pathogenesis of *EGFR* mutation in lung cancer [49].

Despite the evidence presented, we should bear in mind is that cases available for the current study (both the meta-analysis and the following dataset curation) are very limited. Caution is warranted in the data interpretation. Moreover, lung cancer is multifactorial and the genetics basis is complex. Current research on cancer predisposing genes is usually based on assumptions, which would over-extrapolate the data [16]. Many susceptibility genes may only explain a small portion of the inherited susceptibility; but these genes with small or moderate effects might, in combination, act additively or synergistically to result in lung cancer susceptibility. The acquisition of specific somatic mutations in a background of predisposing genes may drive cancer evolution in a particular direction. Which genes behave this way and how the genetic aberrations function during lung cancer evolution are still undetermined.

Regarding the current study, other drawbacks besides data limitation include: 1) recall bias and selective reporting bias due to retrospective study designs; 2) mostly Asian patients, which are possibly not representative of other ethnicities; 3) heterogeneity in detection methods [85]; 4) intra-tumour heterogeneity (one single diagnostic assessment may not represent the whole picture) [79]; 5) differences in definitions regarding EGFR positive mutation (however, we presume the conclusion would not be significantly changed, since L858R and 19del are the most frequently mutated in lung cancer and other mutations reported in the studies we pooled here are limited); 6) heterogeneity in study populations (subgroup analysis in the current meta-analysis may help); 7) bias resulting from self-reported family history (However, this may not be a major issue, since there is a high positive predictive value and sensitivity in it by a recent systematic review [86]).

## Conclusions

Given current evidence and our observations, there are potentially different genetic modifiers in somatically *EGFR*-mutant lung cancers from their wild-type counterparts. Familial lung cancers tentatively favour adenocarcinoma, females, never-smokers, coexistence with secondary somatic *EGFR* mutation and occasionally multi-focal lesions. Among them, germline *EGFR* mutation carriers affected with lung cancers are more frequently the White ethnicity. Some mechanisms such as energy balance may attribute to the specific secondary *EGFR* mutation type in the tumour of familial cases. However, caution needs to be taken when interpreting the data, as it is incomplete. Further studies on this topic should be encouraged, which will hopefully provide a more detailed genetic landscape for lung cancer aetiology.

## Supplementary information

**Additional file 1: Table S1.** Searching strategies for the meta-analysis. **Table S2.** Searching strategies for the second literature review. **Table S3.** Evaluation of case-control study quality with The Newcastle-Ottawa Scale (NOS) in meta-analyses. **Table S4.** Evaluation of cohort study quality with The Newcastle-Ottawa Scale (NOS) in meta-analyses. **Figure S1.** Forest plot of family history of any cancer and the risk of EGFR positive mutation (11 studies included). **Figure S2.** Funnel plot of family history of cancer and the risk of somatic EGFR positive mutation in lung cancer.

## Data Availability

All data generated or analysed during this study are included in this published article and its supplemental information files.

## Abbreviations

ADC: Adenocarcinoma
CPG: Cancer predisposition gene
EGFR: Epidermal growth factor receptor
FH_All: Family history of all cancers
FH_Any: Family history of any cancer
FH_Other: Family history of other cancer except lung cancer
FHLC: Family history of lung cancer
NSCLC: Non-small cell lung cancer
OR: Odds ratio
TKI: Tyrosine kinase inhibitor
