# Supplementary material for: Potential genetic modifiers for somatic EGFR mutation in lung cancer: a meta-analysis and literature review
Source: BMC Cancer. 2019 Nov 8;19:1068. doi: 10.1186/s12885-019-6317-6 (PMC6842246; doi:10.1186/s12885-019-6317-6)
Supplement: Supplementary file 1 — Additional file 1: Table S1. Searching strategies for the meta-analysis. Table S2. Searching strategies for the second literature review. Table S3. Evaluation of case-control study quality with The Newcastle-Ottawa Scale (NOS) in meta-analyses. Table S4. Evaluation of cohort study quality with The Newcastle-Ottawa Scale (NOS) in meta-analyses. Figure S1. Forest plot of family history of any cancer and the risk of EGFR positive mutation (11 studies included). Figure S2. Funnel plot of family history of cancer and the risk of somatic EGFR positive mutation in lung cancer. [file 12885_2019_6317_MOESM1_ESM.docx]

**Additional Files**

**Potential genetic modifiers for somatic EGFR mutation in lung cancer: a meta-analysis and literature review**

Yue I. Cheng ^1,2,§^, Yuncui Gan ^1,§^, Dan Liu ^1^, Michael P.A. Davies ^2^, Weimin Li ^1,*^, John K. Field ^2^

^1^ Department of Respiratory and Critical Care Medicine, West China Hospital, Sichuan University, Chengdu, 610041, China

^2^ Lung Cancer Research Group, Department of Molecular and Clinical Cancer Medicine, Institute of Translational Medicine, University of Liverpool, William Henry Duncan Building, 6 West Derby Street, Liverpool L7 8TX, United Kingdom.

* Corresponding author: Weimin Li, E-mail: [weimin003@163.com](mailto:weimin003@163.com).

§ The two authors contribute equally to the paper.

**Tables**

**Table S1 Searching strategies for the meta-analysis**

| **Database** | **Query (as of 26^th^ July 2018)** | **Items found** |
| --- | --- | --- |
| **PubMed** | (((((((((((((((((((((((((((((("Genes, erbB-1"[Mesh]) OR Genes, erbB1) OR Gene, erbB1) OR erbB1 Gene) OR erbB1 Genes) OR erbB-1 Genes) OR erbB 1 Genes) OR erbB-1 Gene) OR v-erbB Genes) OR v erbB Genes) OR v-erbB Gene) OR v-erbB Oncogenes) OR v erbB Oncogenes) OR v-erbB Oncogene) OR c-erbB-1 Genes) OR c erbB 1 Genes) OR c-erbB-1 Gene) OR EGFR Genes) OR Epidermal Growth Factor Receptor Genes) OR c-erbB-1 Proto-Oncogenes) OR c erbB 1 Proto Oncogenes) OR c-erbB-1 Proto-Oncogene) OR Genes, EGFR) OR EGFR Gene)) AND (((((((((((((((((("Lung Neoplasms"[Mesh]) OR Pulmonary Neoplasms) OR Neoplasms, Lung) OR Lung Neoplasm) OR Neoplasm, Lung) OR Neoplasms, Pulmonary) OR Neoplasm, Pulmonary) OR Pulmonary Neoplasm) OR Lung Cancer) OR Cancer, Lung) OR Cancers, Lung) OR Lung Cancers) OR Pulmonary Cancer) OR Cancer, Pulmonary) OR Cancers, Pulmonary) OR Pulmonary Cancers) OR Cancer of the Lung) OR Cancer of Lung))))) AND ((("Medical History Taking"[Mesh]) OR familial) OR family history) | 385 |
| **Web of Science (All databases)** | #1 TS=(lung tumour* OR lung tumor* OR pulmonary tumor* OR pulmonary tumour* OR lung cancer* OR pulmonary cancer*)  #2 TS=(erbB1 Gene* OR erbB-1 Gene* OR erbB 1 Gene* OR v-erbB Gene* OR v erbB Gene* OR v-erbB Gene* OR v-erbB Oncogene* OR v erbB Oncogene* OR c-erbB-1 Gene* OR c erbB 1 Gene* OR c-erbB-1 Gene* OR EGFR Gene* OR Epidermal Growth Factor Receptor Genes OR c-erbB-1 Proto-Oncogene* OR c erbB 1 Proto Oncogene*)  #3 TS=(family histor* OR familial)  #4 #1 AND #2 AND #3 | 120 |
| **EmBase** | #1 'lung cancer'/exp OR 'lung cancer'  #2 'epidermal growth factor receptor'/exp OR 'epidermal growth factor receptor'  #3 'family history'/exp OR 'family history' OR familial OR 'familial disease'/exp OR 'familial disease'  #4 #1 AND #2 AND #3 | 233 |
| **Cochrane Library** | #1 MeSH descriptor: [Lung Neoplasms] explode all trees  #2 pulmonary near cancer* or pulmonary near neoplasm* or pulmonary near carcinoma* or pulmonary near tumour* or pulmonary near tumor* (Word variations have been searched)  #3 lung near cancer* or lung near neoplasm* or lung near carcinoma* or lung near tumour* or lung near tumor* (Word variations have been searched)  #4 #1 or #2 or #3  #5 MeSH descriptor: [Genes, erbB-1] explode all trees  #6 erbB1 or v-erbB or erbB-1 or erbB near 1 or c-erbB-1 (Word variations have been searched)  #7 "EGFR" or "epidermal growth factor receptor" (Word variations have been searched)  #8 #5 or #6 or #7  #9 MeSH descriptor: [Medical History Taking] explode all trees  #10 family near histor* or "familial aggregation" or "familial" or inherit* or heredita* (Word variations have been searched)  #11 #9 or #10  #12 #4 and #8 and #11 | 11 |

**Table S2** **Searching strategies for the literature review**

| **Database** | **Query (as of 28^th^ Dec 2018)** | **Items found** |
| --- | --- | --- |
| **PubMed** | ((((((((family history[Text Word]) OR familial[Text Word])) OR "Family"[Mesh]) OR "Heredity"[Mesh]) OR "Pedigree"[Mesh])) AND ((((((((germline mutation[Text Word]) OR germ line mutation[Text Word]) OR predispos* gene[Text Word]) OR germ-line mutation[Text Word])) OR "Genetic Predisposition to Disease"[Mesh]) OR "Germ-Line Mutation"[Mesh]) OR "Genetics"[Mesh])) AND ((lung cancer[Text Word]) OR "Lung Neoplasms"[Mesh]) | 315 |
| **Web of Science Core Collection** | #1 TOPIC: (lung cancer)  #2 TOPIC: (familial lung cancer) OR TOPIC: (family history) OR TOPIC: (heredity) OR TOPIC: (pedigree)  #3 TOPIC: (germline mutation) OR TOPIC: (germ line mutation) OR TOPIC: (Predispos* gene) OR TOPIC: (gene*) OR TOPIC: (germ-line mutation)  #4 #3 AND #2 AND #1  #5 TITLE: (lung) OR TITLE: (pulmonary)  #6 #5 AND #4 | 431 |

**Table S3 Evaluation of case-control study quality with The Newcastle-Ottawa Scale (NOS) in meta-analyses.**

| **NEWCASTLE - OTTAWA QUALITY ASSESSMENT SCALE**  **- CASE CONTROL STUDIES** | | Cheng et al, 2015 |
| --- | --- | --- |
| Selection | **1) Is the case definition adequate?**  a) yes, with independent validation *  b) yes, e.g. record linkage or based on self-reports  c) no description | b |
|  | **2) Representativeness of the cases**  a) consecutive or obviously representative series of cases *  b) potential for selection biases or not stated | a |
|  | **3) Selection of Controls**  a) community controls *  b) hospital controls  c) no description | a |
|  | **4) Definition of Controls**  a) no history of disease (endpoint) *  b) no description of source | a |
| Comparability | **1) Comparability of cases and controls on the basis of the design or analysis**  a) Study controls for lung cancer subtypes (e.g. non-small cell lung cancer) *  b) Study controls for any additional factor * (e.g. never-smoking.) | ab |
| Exposure | **1) Ascertainment of exposure**  a) secure record (e.g. surgical records) *  b) structured interview where blind to case/control status *  c) interview not blinded to case/control status  d) written self-report or medical record only  e) no description | a |
|  | **2) Same method of ascertainment for cases and controls**  a) yes *  b) no | a |
|  | **3) Non-Response rate**  a) same rate for both groups *  b) non respondents described  c) rate different and no designation | c |

Items earning stars were yellow-coloured.

**Table S4 Evaluation of cohort study quality with The Newcastle-Ottawa Scale (NOS) in meta-analyses.**

| **NEWCASTLE - OTTAWA QUALITY ASSESSMENT SCALE - COHORT STUDIES** | | Gaughan et al, 2013 | He et al, 2013 | Hsu et al, 2016 | Isla et al, 2016 | Kawaguchi et al, 2011 | Kim JS et al, 2017 | Kim SY et al, 2017 | Okudela et al, 2009 | Wang et al, 2015 | Zhu et al, 2014 |
| --- | --- | --- | --- | --- | --- | --- | --- | --- | --- | --- | --- |
| Selection | 1)  **Representativeness of the exposed cohort:**  a) truly representative of the average lung cancer patients in the community*;  b) somewhat representative of the average lung cancer patients in the community* (generally somewhat selected due to inclusion and/or exclusion criteria without detailed description of the excluded data, i.e. only patients with known EGFR mutation status and family history of cancer were included);  c) selected group of users e.g. nurses, volunteers;  d) no description of the derivation of the cohort | **b** | **b** | **b** | **a** | **b** | **a** | **b** | **b** | **b** | **b** |
|  | **2) Selection of the non-exposed cohort:**  a) drawn from the same community as the exposed cohort*;  b) drawn from a different source;  c) no description of the derivation of the non-exposed cohort | **a** | **a** | **a** | **a** | **a** | **a** | **a** | **a** | **a** | **a** |
|  | **3) Ascertainment of exposure:**  a) secure record (e.g. surgical records)*;  b) structured interview*;  c) written self-report (generally self-reports or physician notes in the medical records);  d) no description | **c** | **c** | **c** | **b** | **b** | **b** | **c** | **b** | **c** | **c** |
|  | **4) Demonstration that outcome of interest was not present at start of study:**  a) yes*;  b) no | **a** | **a** | **a** | **a** | **a** | **a** | **a** | **a** | **a** | **a** |
| **Comparability** | **1) Comparability of cohorts on the basis of the design or analysis:**  a) study controls for lung cancer subtypes (e.g. non-small cell lung cancer, lung adenocarcinoma)*;  b) study controls for other factors (e.g. never-smoking, female)* | **ab** | **a** | **a** | **b** | **ab** | **a** | **ab** | **a** | **a** | **a** |
| **Outcome** | **1) Assessment of outcome:**  a) independent blind assessment*;  b) record linkage*;  c) self-report;  d) no description | **a** | **a** | **a** | **a** | **a** | **a** | **a** | **a** | **a** | **a** |
|  | **2) Was follow-up long enough for outcomes to occur:**  a) yes*;  b) no | **a** | **a** | **a** | **a** | **a** | **a** | **a** | **a** | **a** | **a** |
|  | **3) Adequacy of follow up of cohorts:**  a) complete follow up - all subjects accounted for*;  b) subjects lost to follow up unlikely to introduce bias - less than 20 % lost or description of those lost suggested no difference from those followed*;  c) follow up rate < 80% and no description of those lost;  d) no statement (generally no statement of the missing outcome data – EGFR detection rate - in the study cohort due to that patients without EGFR mutation status would be excluded when selecting patients) | **d** | **d** | **a** | **b** | **b** | **b** | **d** | **a** | **d** | **a** |

Items earning stars were yellow-coloured.

**Figures**

**
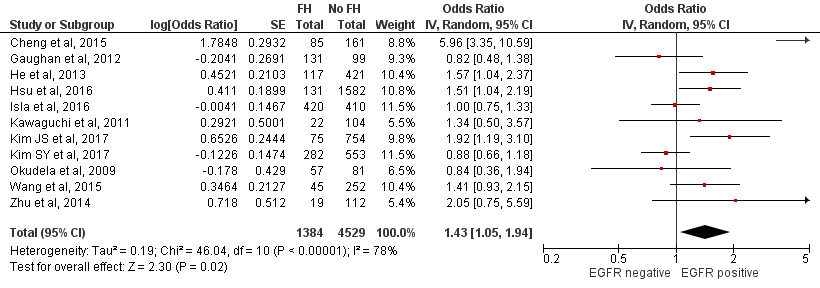
**

**Figure S1 Forest plot of family history of any cancer and the risk of EGFR positive mutation (eleven studies included).** FH, family history; IV, Inverse Variance method CI, confidence interval.

**
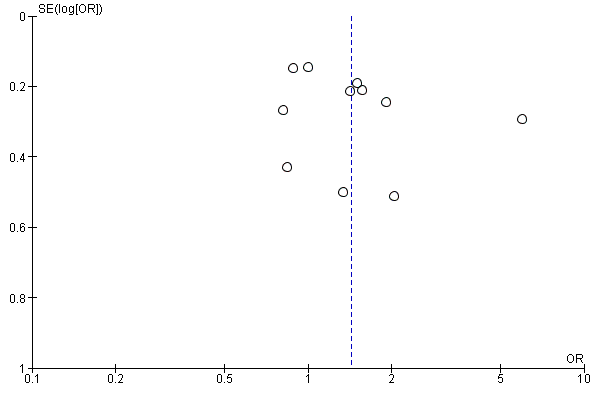
**

**Figure S2 Funnel plot of family history of cancer and the risk of somatic EGFR positive mutation in lung cancer.**
